# Pauperism and Crime

**Published:** 1910-08-13

**Authors:** 


### PAUPERISM AND CRIME.

There are two departments of the State in which hitherto no success has been secured, and which would be all the better for establishment on a thoroughly sound and scientific basis. The first of these is our penal department. Modern views on crime are happily becoming more sane, and we are endeavouring to shake off that baneful notion of moral responsibility which, dating from early Judaism, was cultivated to such an extent during the Victorian era. Because we can recognise that crime may in many cases be a disease, or the result of unfavourable circumstances, it does not in any way mitigate our treatment of the criminal. Because we have discovered that rabies is a microbic disease, and ceased to look upon it as possession by a devil, it does not lessen our efforts to stop and repress it. The charge of our prisons is very frequently, if not always, given to retired military men. This procedure is self-stultification. Until recently our military system was synonymous with incompetence. Rarely did the mind of the officer arrive at an understanding of his own business, nor was he ever likely to acquire any deep knowledge of human nature, scientifically grounded, such as is requisite in dealing with the criminal. A system which avowedly turns men into unthinking automatons was thus applied without discrimination to reform or punish the criminal. Revenge and retaliation, cast-iron rules such as are engendered by long acquaintance with pipe-clay were, and in many still are, the order of the day. That, as we have already said, is attempting to reform at the wrong end. The head of a large prison should be a medical man, assisted by a competent staff of mixed medical and lay members. No amount of trouble should be spared in endeavouring to make the criminal useful. We have the example of the German penal settlements, which certainly demonstrate that the man who will not work according to ordinary principles has to be made to do so. Nor again would the barbarous and wasteful method of hanging be employed. If it is decided that a man can be of no use as a normal citizen, he should be dealt with specially in the best interests of the community.

Just in the same way our methods of pauper relief and the question of unemployment need to be dealt with by competent medical men. Many people seem to forget that after an absence of food for some days a man loses "morale," and the first thing to do is not to send him out to work in his then condition, but to feed him, rest him, and restore his health. Then if he will not work he should be forced to do so. Unemployed are rendered unemployable daily simply through the want of forethought of those who deal with the question.

These positions, which involve responsibilities and necessitate a large experience of human nature, should be highly paid, as being perhaps the most useful from a citizen's point of view, and the occupants should undergo not academical examinations but an inquiry into their previous career, to see if their experience has been wide enough. This will tend to exclude satisfactorily the purely scientific doctor, whose position is rightly in the laboratory. But that great advantage would accrue from such a reform cannot be doubted for a moment. We want a national basis for our State. We want to get rid on the one side of military inelasticity and on the other to diminish as much as possible exaggerated legal formality. In this country we are beridden by men whose intelligence, though excellent, is incapable of grasping any problem which does not offer a verbal quibble for solution.

